# Complications of Otitis Media: Mastoiditis and an Intracranial Abscess Presenting as Behavioral Changes

**DOI:** 10.7759/cureus.111924

**Published:** 2026-07-01

**Authors:** Tamer Yahya, Emily C Barbee, Ameer Kittaneh

**Affiliations:** 1 Department of Emergency Medicine, Mercy Health - St. Rita's Medical Center, Lima, USA; 2 Division of Human Genetics and James Comprehensive Cancer Center, The Ohio State University Wexner Medical Center, Columbus, USA

**Keywords:** abscess, behavioral changes, complications, mastoiditis, otitis

## Abstract

An intracranial abscess is a rare but life-threatening complication of otitis media (OM) and mastoiditis that requires rapid diagnosis and intervention. We present the case of a 43-year-old woman who presented with acute altered mental status and aggressive behavior following recent treatment for OM. Physical examination revealed a perforated, purulent right tympanic membrane, and neuroimaging confirmed bilateral mastoiditis and a right temporal lobe brain abscess with midline shift. Following treatment with broad-spectrum intravenous antibiotics, the patient underwent a successful craniotomy for abscess evacuation, a mastoidectomy, and tympanostomy tube placement. Postoperatively, her behavioral and neurological symptoms fully resolved, and she was discharged to complete a prolonged course of antimicrobial therapy. This case underscores the importance of maintaining a high index of suspicion for otogenic intracranial complications in patients presenting with acute behavioral changes, emphasizing that early, multidisciplinary surgical and medical management is essential to optimize patient outcomes.

## Introduction

Otitis media (OM) can be complicated by hearing loss, mastoiditis, cholesteatoma, meningitis, facial nerve paralysis, and, rarely, brain intracranial and extracranial abscesses [1]. The incidence of brain abscess has been rising over recent decades, from 0.60 to 0.90 per 100,000 in Denmark (1982-2016) and from 1.24 to 2.86 per 100,000 in England (1999-2019), driven largely by aging populations and increasing immunosuppression [2]. Intracranial complications of OM have historically been a significant cause of mortality, particularly in countries with limited access to health education and care [3]. A large-scale study conducted from 1978 to 1990 tracking 24,321 OM patients revealed that 0.36% suffered from intracranial complications, resulting in a mortality rate of 18.4% among those affected [4]. More recently, these complications have been less frequently observed in practice due to the increasing use of antibiotics [5].

Mastoiditis is most commonly caused by bacteria from middle ear infections [6]. Due to the mastoid bone’s proximity to the brain, untreated infection poses a risk for major sequelae, including hearing loss and intracranial extension [7]. Symptoms can include severe headache, fever, neurological deficits, and behavioral changes. We present a case of a 43-year-old woman with a recent history of OM who presented to the emergency department (ED) with behavioral changes and was diagnosed with concurrent mastoiditis and an intracranial abscess.

## Case presentation

ED visit

A 43-year-old woman with a past medical history of developmental delay presented to the ED accompanied by her family due to altered mental status (AMS). The patient exhibited aggressive behavior and was described as acting strangely, cursing at family members, which was uncharacteristic.

For six days prior to the ED visit, the patient had been complaining of right ear pain and fever. She was seen at urgent care three days before her ED visit and was prescribed amoxicillin for OM. Purulent drainage from the right ear was noted in addition to bilateral tympanic membrane (TM) bulging per the urgent care note. Her symptoms persisted, and she reported feeling unwell, stopped going to work, and had poor sleep.

Two days prior to ED presentation, the patient’s mother noted the patient to have alteration in her mental status described as confusion. EMS was called, but the patient refused to come to the ED for evaluation. Thereafter, the patient’s symptoms seemed to improve. She continued taking the antibiotic and was no longer confused or febrile.

On the morning of the day of presentation, the patient’s symptoms worsened with behavioral changes described as being aggressive and using profanity towards her family. The patient denied having any more fever or chills. She did report persistent ear pain and hearing loss on the right side. On review of systems, increased thirst and menorrhagia for the past several months were noted. In addition, it was reported that the patient has not seen a physician in several years.

The physical exam revealed a patient with inappropriate behavior, who was disruptive and non-cooperative with the exam. She was alert and oriented to person, time, and place. There was obvious pustular drainage from the right ear with tenderness at the external ear. The right TM was perforated, with pus noted in the external auditory canal. No mastoid swelling, tenderness, or redness was noted. The left ear had cerumen impaction, and the TM was difficult to visualize. Pupils were equal, round, and reactive to light. The patient was moving all extremities appropriately but was not cooperative enough for a full neurological exam.

The initial vital signs were blood pressure 118/58 mmHg, pulse: 92 bpm, respiratory rate: 30 bpm, oral temperature: 97.7 F, and SpO2: 99% on room air.

The initial labs were significant for the following: mild leukocytosis of 11.2 thou/mm^3^; microcytic anemia with a hemoglobin of 5.6 gm/dl, MCV 59.4 fL; sodium of 130; platelets 718 thou/mm^3^. Blood cultures from two sites were collected and did not show any growth. Urine drug screen was negative, and lactate was normal at 1.6 mmol/L. Findings are summarized in Table 1. 

**Table 1:** Laboratory Results on Initial Presentation

Test	Result	Reference Range
WBC	11.2 (H)	4.8 - 10.8 thou/mm^3^
Hemoglobin	5.6 (L)	12.0 - 16.0 gm/dl
MCV	59.4 (L)	81.0 - 99.0 fL
Platelets	718 (H)	130 - 400 thou/mm^3^
Sodium	130 (L)	135 - 145 meq/L
Lactate	1.6	0.5 – 2 mmol/L
Blood Culture	No Growth	n/a

During the ED stay and prior to obtaining imaging, the patient became increasingly agitated and ripped off her own IV in an attempt to leave. She was treated with 2mg IM lorazepam and 5mg IM droperidol intramuscularly for agitation and to facilitate obtaining the head computed tomography (CT) scan.

A CT of the head with IV contrast was obtained due to suspected intracranial extension of infection and revealed an abnormal density in the right temporal lobe anteriorly, suspicious for an inflammatory process or an infarct. Additionally, there were inflammatory changes in the mastoid air cells bilaterally and in the right middle ear cavity (Figure 1). A brain MRI was suggested by the radiologist and was ordered for better characterization of the noted lesion.

**Figure 1:**
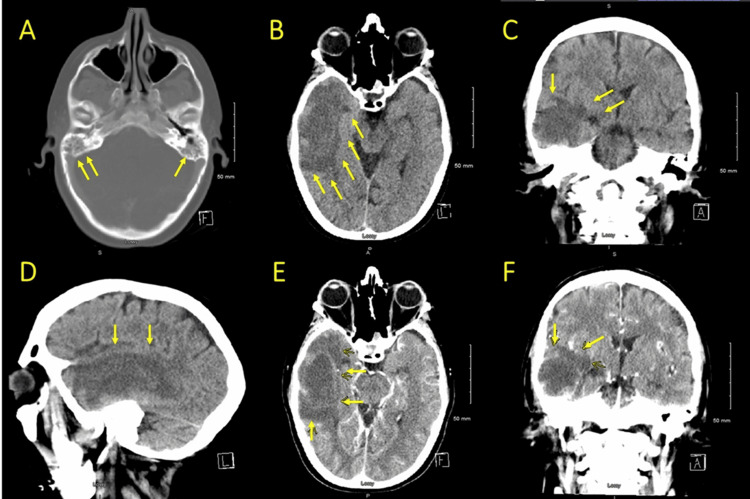
Initial Head CT With and Without Contrast (A) Post-contrast sinus windows showing bilateral mastoiditis, worse on the right side with right side. There is extensive opacification in the mastoid air cells bilaterally and in the right middle ear cavity, consistent with inflammatory changes. (B-D) Pre-contrast axial, coronal, and sagittal images showing ill-defined hypodensity in the right temporal lobe. (E and F) Post-contrast axial and coronal images showing some ring enhancement.

The patient was given ceftriaxone, vancomycin, and acyclovir IV in the ED before being admitted.

Initial hospitalization course 

Brain MRI with and without contrast revealed a 5.8 x 3.0 x 3.2 cm intra-axial mass in the right temporal lobe with rim enhancement and moderate adjacent vasogenic edema, consistent with an abscess. Additional findings included bilateral mastoiditis and a 2 mm leftward midline shift (Figure 2).

**Figure 2:**
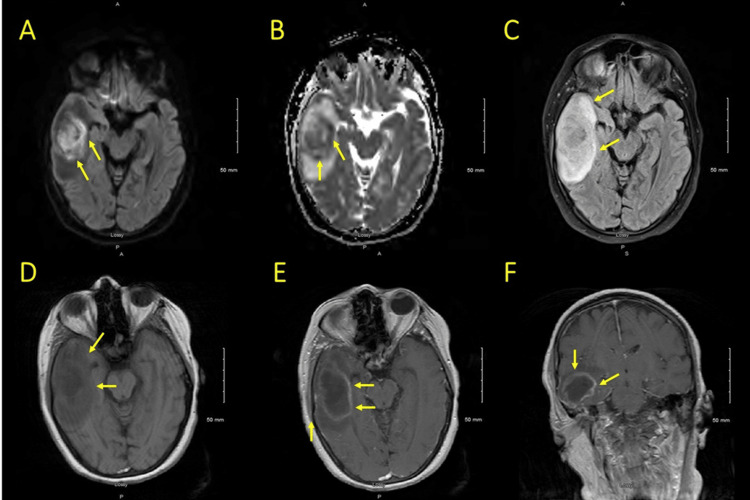
Brain MRI With and Without Contrast (A and B) DWI and ADC windows demonstrating an area of restricted diffusion with a high signal on DWI and a low signal on ADC in the right temporal lobe. (C) The FLAIR window showing FLAIR hypersignal indicating vasogenic edema (involving the white matter and preserving the cortex). (D) Pre-contrast T1 axial image. (E and F) Post-contrast T1 showing a rim-enhancing 5.8 x 3.0 x 3.2 cm intra-axial mass involving the right temporal lobe. DWI: Diffusion-weighted imaging; ADC: apparent diffusion coefficient; FLAIR: fluid-attenuated inversion recovery

Shortly after hospitalization, the patient’s mental status changed, with a decrease in her Glasgow Coma Scale (GCS) from 14 to 12. She was treated with 10mg IV dexamethasone, 1000mg IV levetiracetam, and 12.5 g of IV mannitol. Additionally, 2g of IV Ampicillin was given, and a second head CT was obtained. The second head CT did not reveal any changes from the previous head CT. Transfusion of two units of packed red blood cells for anemia was performed. The process for transport was initiated to a tertiary center with a higher level of care for intracranial intervention, as was recommended by consulting services.

Outside the hospitalization course

On the second day of hospitalization, the patient arrived at the tertiary center with continued AMS. She was described as combative, talking incoherently, and verbally abusive to staff and others. Her physical exam was unchanged. She was admitted to the neuroscience intensive care unit (NICU), where antibiotics were continued with IV metronidazole added. Consults were placed to neurosurgery, otolaryngology, infectious disease, neurology, and gynecology. A continuous EEG (electroencephalogram) was placed, and IV levetiracetam was switched to 50mg of IV brivaracetam BID. She was taken to the operating room for a middle fossa craniotomy for abscess evacuation and external ventricular drain (EVD) insertion, mastoidectomy, and placement of bilateral pressure-equalizing tubes by the neurosurgeon. The patient remained intubated post-operatively and was extubated on the third day of hospitalization. The infectious disease physician agreed with the current antibiotic regimen pending culture results. The EEG obtained did not show any electrographic seizures or non-convulsive status epilepticus.

On the fourth day of hospitalization, the patient was continued on antibiotics with intraoperative cultures remaining negative. She was transferred to the medical floor. Her mental status was noted to be improved, with less agitation and more appropriate behavior. On the following day, the EVD was discontinued. The patient was then discharged, having returned to baseline functional and mental status, with a six-to-eight-week course of IV metronidazole and ceftriaxone, ciprofloxacin-dexamethasone ear drops, a dexamethasone taper, and seizure prophylaxis with brivaracetam for seven days. Operative cultures were all negative but were notably obtained after IV antibiotics had been initiated. An MRI was repeated 11 days after initial presentation (Figure 3). 

**Figure 3:**
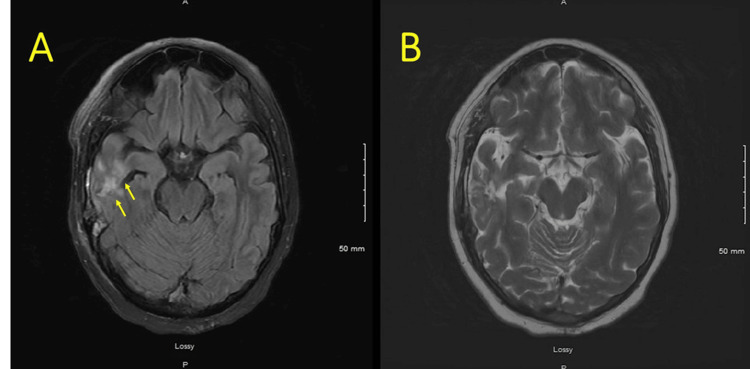
Follow-up Brain MRI without Contrast Post-intervention and treatment MRI. (A and B) FLAIR and T2 images demonstrating the operative site in the right temporal lobe with a surrounding high signal. The surrounding high signal can represent some residual edema versus developing encephalomalacia. The abscess has been surgically resected. No evidence of restricted diffusion (not shown here). FLAIR: Fluid-attenuated inversion recovery

## Discussion

A brain abscess is a confined collection of necrotic tissue located in the brain parenchyma, typically stemming from surgical interventions, traumatic injuries, or microbial infections. Rather than originating in the brain itself, these lesions frequently develop when pathogens migrate from nearby anatomical sites, including the paranasal sinuses, teeth, middle ear, epidural space, or mastoid air cells, with otitis media and mastoiditis specifically associated with abscesses in the inferior temporal lobe. While an intracranial abscess can affect individuals across any age group, clinical data show a peak incidence among adults in the third decade of life. Effectively managing a brain abscess requires a tailored approach combining both pharmaceutical therapies and surgical drainage, with the exact strategy dictated by the lesion's overall size and location [8,9].

Intracranial abscesses exhibit a strong gender disparity, occurring two to three times more frequently in men than in women. The median age is 30-40 years, and morbidity is highest in the fourth decade of life [10]. Associated neurological complications are highly prevalent, characterized by focal motor deficits, lingering AMS, and seizures in up to 80% of patients [11]. Epidemiologically, these lesions constitute about 8% of all intracranial masses in developing regions but only up to 2% in Western countries [12]. Ultimately, prognosis depends heavily on age and initial neurological health; delayed hospitalization, preexisting focal deficits, compromised immunity, uncontrolled diabetes, and a GCS score below 12 are all major drivers of mortality and permanent disability [13].

The clinical features of a brain abscess are highly variable, determined by the primary source of infection alongside the size, number, location, and specific neurological structures affected by the lesions [14]. Headache is the most common presenting symptom and classically worsens at night when a supine position increases intracranial pressure [15]. About half of patients experience focal neurological signs and symptoms [16]. Depending upon the abscess location, manifestations can include visual deficits, dysarthria, hemiparesis, or personality changes, the latter particularly common with frontal or temporal involvement. New-onset seizure may be the presenting symptom in 25% of cases [15]. Signs of intracranial hypertension, including vomiting, nausea, and AMS, are frequently observed. Only about half of patients display fever; it is often low-grade or absent [17]. Nathoo et al. reported that the duration of symptoms can range from one day to eight weeks [18]. Leukocytosis is identified in approximately half of patients, and C-reactive protein is elevated in roughly 90% [19]. Two blood cultures should be performed initially, with further cultures obtained following surgery, as surgical manipulation could provoke bacteremia. Blood cultures may be positive in about 25% of patients [20].

Diagnostic confirmation relies on neuroimaging, which frequently begins with a contrast-enhanced CT scan. On both CT and MRI, these lesions characteristically manifest as a hypodense core surrounded by a distinct, contrast-enhancing ring [11]. Imaging also determines the lesion's size, number, stage, character, and location, while simultaneously assessing for edema, hydrocephalus, or concurrent infections like ventriculitis and mastoiditis to shape the treatment strategy. MRI is the gold standard for imaging of brain abscesses. Compared to a CT scan, it offers superior visualization of early-stage cerebritis and posterior fossa lesions, with diffusion-weighted imaging yielding sensitivity and specificity rates that exceed 95% [21]. Notably, an intracranial abscess stands out as the only CNS infection where performing a lumbar puncture is not recommended, as it offers no diagnostic utility for this condition and increases the likelihood of herniation [22].

Achieving optimal outcomes in cases of intracranial abscess demands an integrated, multi-specialty approach. Generally, neurosurgery works in close association with an infectious disease specialist, intensivist, neurologist, and, if needed based upon etiology, otolaryngology or other specialties. In clinically stable patients scheduled for imminent operative intervention, holding antibiotic administration is preferred to maximize the diagnostic yield of intraoperative cultures. Otherwise, empiric antimicrobials should be initiated immediately post-blood culture collection [15]. 

About 50% of intracranial abscesses are due to *Streptococcus *species, 25% are due to anaerobes, with the remainder due to *Staphylococcus*, *Enterobacteriaceae*, *Nocardia*, and others [15]. Initial empiric choices require broad-spectrum medications that efficiently cross the blood-brain and blood-CSF barriers and should include coverage for anaerobes. Standard regimens combine metronidazole with a third-generation cephalosporin, adding vancomycin if the patient's history includes recent neurosurgery or penetrating head trauma [11]. Intracranial abscesses may be polymicrobial in up to 50% of cases, especially in those with contiguous extension. Due to their polymicrobial nature, identifying one specific microbe does not rule out the presence of others. Therefore, broad-spectrum coverage must be maintained even after a single pathogen is isolated [15]. This is also partly because the techniques used for obtaining cultures are often suboptimal in anaerobe isolation, so coverage for anaerobic organisms should be continued [10]. Ultimately, the total duration of antimicrobial therapy must be highly individualized, calculated using the lesion's size, the choice of surgical approach, the specific microbial profile, and the patient's ongoing clinical response. Long-term, longitudinal outpatient follow-up with an infectious disease specialist is mandatory to oversee the regimen. A six-week duration of therapy is often utilized for surgically treated abscesses [14].

The decision to pursue surgical drainage or stereotactic aspiration versus empiric medical therapy without any intervention depends upon several factors. Potential indications for drainage include the need to obtain culture material, especially in immunosuppressed patients. Stereotactic aspiration allows sampling of small abscesses 1 cm or larger in size [20]. Indications to consider therapeutic drainage include abscesses 2.5 cm or larger in diameter, those exerting mass effect on surrounding tissues, location in the posterior fossa or adjacent to a ventricular surface, or refractory to medical management [15]. Data from Nathoo et al. closely mirror retrospective findings out of Asia and Europe, demonstrating that combining surgical drainage with targeted antimicrobial therapy for brain abscesses exceeding 2.5 cm yields an 81% rate of good clinical outcomes, a 5% rate of poor outcomes, and a 13% mortality rate [18]. When managing associated cerebral edema, the use of corticosteroids remains a subject of debate due to a lack of well-controlled, randomized clinical trials. Corticosteroids are generally recommended perioperatively to reduce intracranial pressure and avoid herniation [23]. Prophylactic anticonvulsant therapy presents another clinical gray area. Long-term tracking by Legg et al. spanning up to three decades revealed that nearly 70% of post-abscess patients eventually experience seizures [24]. Current consensus advises that the length of antiepileptic therapy should be custom-tailored to the individual and monitored via serial electroencephalogram evaluations, with most experts recommending a minimum of three months of prophylaxis provided the patient remains seizure-free [25].

## Conclusions

An intracranial abscess is a very serious and rare condition where a collection of necrosis forms within the brain or surrounding tissues. This can be contiguous, due to the spread of infection from the middle ear and the mastoid bone. Symptoms can include severe headache, fever, neurological deficits, and behavioral changes, which include AMS ranging from confusion to coma, personality alterations, and cognitive deficits. While the clinical incidence is low, physicians should maintain a high index of suspicion for intracranial infections in the presence of AMS and OM.
